# Data on global expression of non-coding RNome in mice gastrocnemius muscle exposed to jararhagin, snake venom metalloproteinase

**DOI:** 10.1016/j.dib.2016.09.052

**Published:** 2016-10-11

**Authors:** Patricia Bianca Clissa, Rodrigo Pessôa, Karla Fernanda Ferraz, Daniela Raguer Valadão de Souza, Sabri Saeed Sanabani

**Affiliations:** a Immunopathology Laboratory, Butantan Institute, Sao Paulo, Brazil; b Clinical Laboratory, Department of Pathology, LIM 03, Hospital das Clínicas, School of Medicine, University of São Paulo, São Paulo 05403 000, Brazil; c Medical Laboratory investigation of dermatological and immune deficiency syndrome – LIM 56, Faculty of Medicine, University of São Paulo, São Paulo 05403 000, Brazil

**Keywords:** Small RNAs sequences, Deep sequencing, Jararhagin, Inflammation

## Abstract

This article describes the data on the global expression profile of small RNA (smRNAs) molecules in mice gastrocnemius muscle exposed to jararhagin, snake venom metalloproteinase. The data include smRNAs in mice gastrocnemius muscle challenged with Jararhagin (Jar; *n*=4) in the right paw or phosphate-buffered saline (PBS; control; *n*=4) in the left paw. smRNA-Seq libraries were generated after 24 h of exposure to PBS or jararhagin. The expression profiles of smRNAs including microRNA and snoRNA were compared between both groups. The sequencing data from both groups have been uploaded to Zenodo http://dx.doi.org/10.5281/zenodo.56492.

**Specifications Table**

**Table:** 

Subject area	*Biology*
More specific subject area	*Inflammation*
Type of data	*Tables, figures*
How data was acquired	*The raw data was generated using the MiSeq Illumina sequencer and was statistically analyzed and displayed in tabular format.*
Data format	*Raw, analyzed*
Experimental factors	*The samples were collected from mice challenged with jararhagin in the right paw (n=4), or phosphate-buffered saline (PBS as a negative control) in the opposite paw (n=4). Total = 8 gastrocnemius muscle from 4 mice in all.*
Experimental features	*Total RNA including smRNAs were isolated from the gastrocnemius muscle using Trizol phase separation assay and then purified using the miRNeasy Mini Kit (QIAGEN Inc.). After measuring the concentration and checking the quality of the RNA, libraries were subjected to smRNA profiling.*
Data source location	*Sao Paulo, Brazil.*
Data accessibility	*Data is within this article and uploaded to Zenodo*http://dx.doi.org/10.5281/zenodo.56492

**Value of the data**

• The data presented in this study enhance our understanding on the mechanism involved in the control of the pathogenesis for snake venom metalloproteinase-induced inflammation.
• The significantly differentially expressed smRNAs demonstrated in these data have the potential to be chosen as biomarkers for exploitation in therapeutic strategies for inflammation caused by snake venom.
• This data may be also valuable for future studies that aim to similar analyses for evaluation and comparison.

## 1 Data

**Fig. 1:**
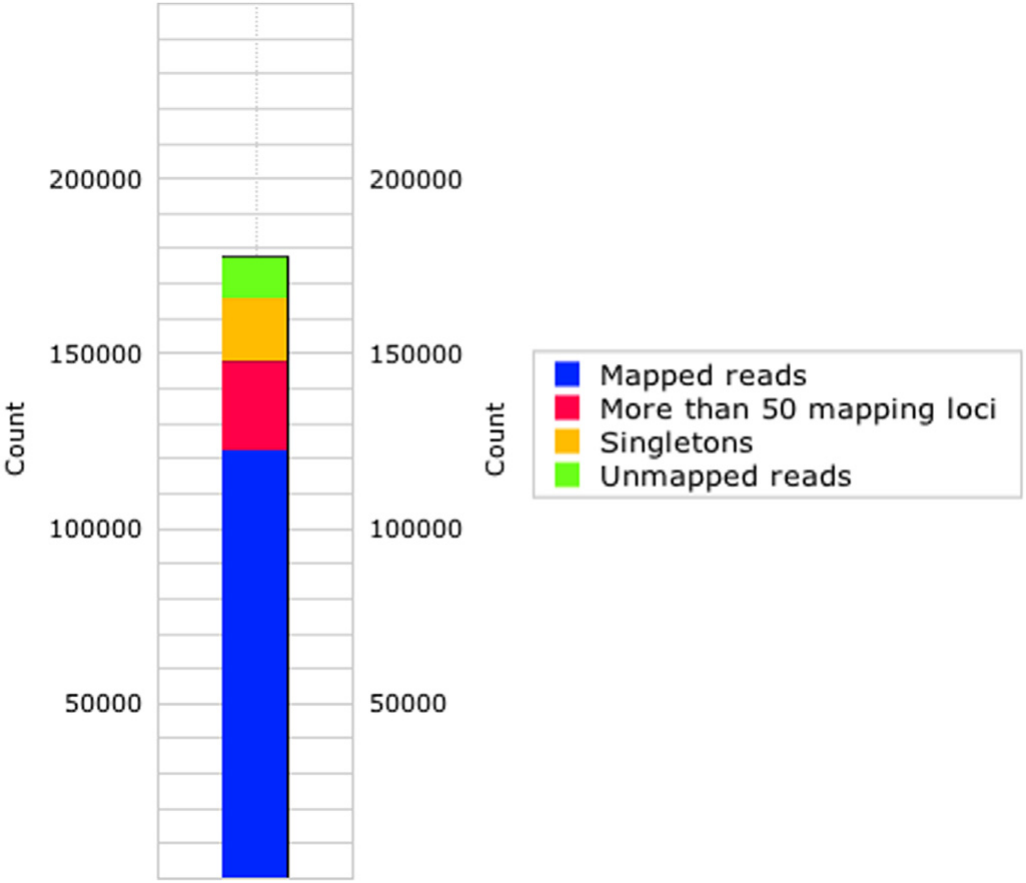
Annotation statistics for the mice gastrocnemius muscle challenged with jararhagin (Jar; *n*=4) in the right paw showing the mapping of reads to the mouse genome.

**Fig. 2:**
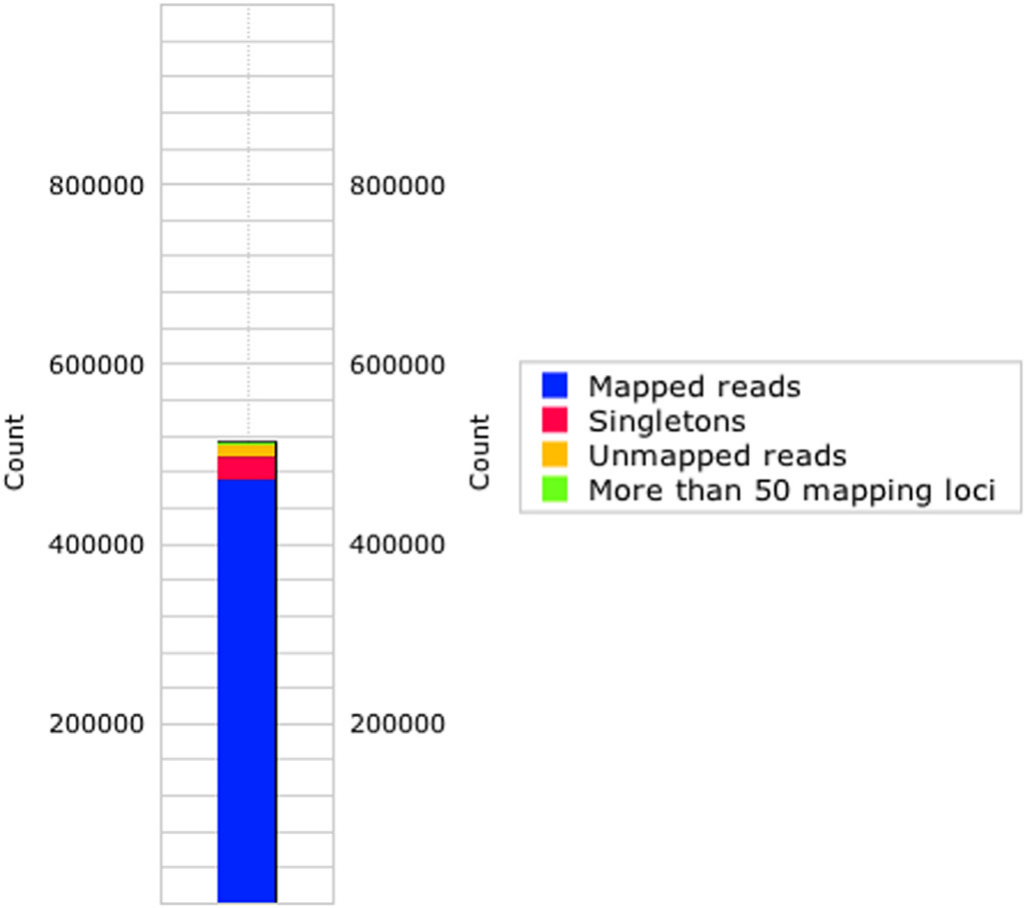
Annotation statistics for the mice gastrocnemius muscle challenged with phosphate-buffered saline (PBS; control; *n*=4) in the left paw showing the mapping of reads to the mouse genome.

The dataset described here consists of partially clean reads of smRNA after removing adaptors. Detailed quantitative information about the smRNA-Seq libraries in PBS and jararhagin groups are depicted in Fig. 1, Fig. 2, respectively. Information about the detailed expression profile and distribution of non-coding RNAs (ncRNA) in the PBS and jararhagin libraries by omiRas (omiRas://tools.genxpro.net/omiras/) are reported in Appendix A, Appendix A, respectively (Supplementary Tables 1 and 2**)**. Pairwise comparisons for all ncRNAs of the PBS versus jararhagin group are displayed in the differential expression profiles in (Supplementary Table 3**)**. The overall data of ncRNA that indicates differential expression with a p value <0.05 are displayed in (Supplementary Table 4**)**.

## 2 Experimental design, materials and methods

### 2.1 Samples

Six- to 8-week-old female Swiss Webster mice were obtained from Butantan Institute. To determine inflammatory response induced by jararhagin, one groups of four mice were challenged intramuscularly (i.m.) into the gastrocnemius muscle with 1 µg of Jararhagin in the right paw (*n*=4) and with phosphate-buffered saline (PBS) in the left paw. After 24 h, mice were scarified and the muscles were dissected, minced, and cells were harvested in TRIzol. Samples were then processed using a miRNeasy Mini kit (Qiagen) according to the manufacturer׳s instructions. The Ethical Committee for Animal Research at the Butantan Institute (CEUAIB), São Paulo, Brazil approved all experiments involving mice (application approval number 8773110516).

### 2.2 RNA preparation

The individual gastrocnemius muscles were homogenized using Trizol according to the manufacturer׳s instructions. RNA was further purified using the miRNeasy Mini kit (Qiagen) following the recommendations of the manufacturer. The purity and quality of RNA samples were verified by spectrophotometry *and* the Agilent 2100 Bioanalyzer, respectively. The assay further confirmed the absence of genomic DNA contamination.

### 2.3 Library construction

For each sample in both groups, smRNA libraries were prepared with the Small RNA v1.5 sample preparation kit as per the manufacturer׳s instructions (Illumina, San Diego, CA). Briefly, 5 μl of purified total RNAs were ligated with 1 μl RNA 3′ Adapter and then with a 5′ RNA adapter (Illumina, San Diego, CA). The 5′ adapter also included the sequencing primer. After RT-PCR amplification, the resulting products were analyzed using polyacrylamide gel electrophoresis (PAGE) (6% Novex Tris-borate-EDTA [TBE] PAGE; Invitrogen). After gel electrophoresis, smRNA bands at sizes 145–150 bp were excised and purified.

### 2.4 Sequencing and read mapping

Each group׳s libraries were sequenced to 60 nt of read length on individual flow cell on a Illumina MiSeq instrument using the MiSeq reagent kits v2. A total of 93.6% of bases had a base quality > Q30 and the real-time error rate of spiked PhiX was 25%. After sequencing, the data were obtained in Illumina FASTQ format (Illumina). The adaptor sequences were first trimmed from smRNA reads. Only smRNA reads with sequences length from 16 to 40 nt were retained for further analysis. For mapping of sequencing output, we used omiRas, a Web server tool detecting all known smRNA sequences annotated in the microRBase registry and predicting novel smRNAs. The differential expression of smRNAs between groups was calculated with the DESeq bioconductor package [1] that considers biological and technical variance into account.
